# Actæa-racemosa

**Published:** 1898-07

**Authors:** C. L. Olds

**Affiliations:** Renovo, Pa.


### ACTÆA-RACEMOSA.

C. L. OLDS, M. D., H. M., RENOVO, PA.

Actæa-racemosa was first used by the Indians as an antidote to snake poisons. It was also used by the squaws to promote easy labor. It got the name of Cimicifuga because it was supposed to keep bugs away (cimex, fugis). The next place we find it is among the eclectics, who used it for rheumatism, and then among the allopaths, who used it for rheumatism and for chorea. The homœopath cures rheumatism and chorea with this remedy, but he uses it only when the symptoms produced by the remedy are present. The homœopath looks to the proving for his knowledge of the drug, the old school to experience; the homœopath has a law upon which to depend, the old school nothing but experience and theory.

Actæa is suitable to women with rheumatism, especially when it is in the belly of the muscles. There is a sore, bruised, lame feeling all over the body. The muscles seem to knot up—cramps come in the calves, in different parts.

The mental state is peculiar; the patient is sad, melancholy, sees no joy in life—feels as if a black cloud or a black pall had settled down over her and weighed like lead upon her heart.

She feels as if she were going crazy. Among other mental symptoms we find fear of death, but unlike the same symptom in Aconite, it is not accompanied by fever. She sees different kinds of animals, rats, mice, and other strange things. She may be taciturn, wanting to be let alone, like Ign., Puls., or may be very voluble, changing rapidly from one subject to another; again, she may be irritable.

Throughout the drug there are neuralgias, in different parts of the body—rheumatic neuralgias—in the eyes, face, scalp. If these are suppressed mania comes on, and she sees animals and fights. When neuralgia or rheumatism leaves mind symptoms come on.

In this remedy are most terrible headaches. There are sensations as if the head were opening or shutting, as if a bolt had been driven up in the back of the head from the neck to the vertex. It seems as if the top of the head would be lifted off. (Cf. Cocc., Bapt., Bell., Cann-ind., and Cann-sat.) She feels as if the brain were too large, a sensation of fullness in the head. The eyes feel full. It seems as if she would go crazy with the headaches. They are worse from motion of any kind, better in the open air. This is the exception in Actæa with regard to cold, the remedy is generally chilly, worse in the open air, but the headaches are better in the open air. They are worse in damp weather, worse from changes of weather, worse from slightest draught, worse on going upstairs. Sometimes the headache begins at the root of the nose, and extends gradually over the head, with delirium and vomiting. With the headaches there is almost always inflammation of the conjunctiva—it looks red and raw.

Actæa is worse in the morning in most complaints. The complaints are chiefly on the left side. Running through the remedy we find numbness in different parts—trembling, shivering. Particularly will these symptoms be found in women—in women with uterine complaints, with hysterical or rheumatic constitutions. We may find affections coming on from disappointed love.

In the eyes we find neuralgia—sharp, sticking pains running into the eyes, as if needles were run in. They are worse at night, and worse on closing the eyes. The eyeballs ache intensely. There is oscillation of the balls, moving to and fro, with great soreness. There is a neuralgia of the malar bone, the pain goes away at night and reappears the next day. There are toothaches in neuralgic patients. There are colicky pains in the abdomen, relieved by bending double. These rheumatic and neuralgic pains are generally relieved by pressure, especially where there are spasms. Whenever the rheumatic or neuralgic affections are suppressed, on will come the mental affections—gloom, despair of life, chorea, mania. When rheumatism ceases, chorea will come on, especially movements of the *left* hand—the left side of the body is more affected.

In rheumatism we may compare this remedy with Actæa-spicata, in which the pains are in the small joints, the hands, fingers, especially the right wrist; in Act-rac. they are in the large muscles. In Act-spi. the joints are intensely sensitive, the least touch will make the patient cry out. The pains are tearing, worse on moving at all, and worse at night. The small joints swell after walking about, either those of the hands or the feet.

Caulophyllum also may be compared in rheumatism. Here there are also affections of the small joints, but not the great sensitiveness. The pains are erratic, move about, like those of Puls. Pains go from one finger to another, and finally to the nape of the neck, here the rheumatic pains centre. They are nearly always associated with uterine troubles, as in Actæa-rac.

The menses are profuse, early, black, offensive, sour, coagulated. Sometimes they are irregular. There is a peculiar thing in Actæa-rac., that although the menses are profuse and early, the more the flow comes on the worse the patient feels, which is the reverse of the case in most remedies. There is, indeed, a general aggravation of the patient during the menstrual flow; the mental symptoms are worse; hysterical or epileptic spasms may occur; the choreic troubles are all worse.

There were forty male provers and only six female. In every one of the females stomach symptoms came out—nausea and vomiting, great retching—but not in one of the males. The remedy cures nausea in pregnancy, also false labor pains occurring during pregnancy.

It is said that if this remedy be given during the last weeks or months of pregnancy it will cause an easy labor. It would be just as plausible to say that it will cure chorea or rheumatism. It will cause an easy labor, but only when the symptoms of the patient correspond to those of the drug. In labor we may find great trembling, shaking, shivering, yet the patient does not feel cold; or there may be chilliness. Pains shoot about in the abdomen, in the broad ligaments, dart from side to side. Pains leave the uterus and go to the hips, and the patient cries out, "Oh, my hips, my hips!" The feeling is as if the hips were seized by something, and yet the patient is relieved if they are seized by the hand. The pains may run down the legs from the hips. The woman is worse from noise, and may have fainting fits.

After-pains come in the groins, in the broad ligaments. These pains in the broad ligaments may be covered by this remedy when occurring at any other time. Puerperal mania may come on, with hysterical convulsions. There are inframammary pains, worse on the left side, and burning in the mammæ.

As a result of suppression of the menses, or of the lochia, mental symptoms come on; the woman feels that a black pall has settled over everything, that there is nothing worth living for. With these mental symptoms there is nearly always sleeplessness—she cannot possibly get to sleep.

If there is heart trouble, pain shoots up to the left shoulder and down the left arm. The left arm will feel numb as if paralyzed, as if bound to the side of the body.

The choreic twitchings and jerkings and irregular motions are all worse in the daytime, and *cease on going to sleep.* They are worse from emotions and during the menstrual periods. They may be caused by suppressed menses, and accompanied by sleeplessness.

In addition to the chronic rheumatism this remedy has inflammatory rheumatism of the large muscles, the belly of the muscles, with intense soreness, sharp, lancinating pains here and there, worse from motion, worse from dampness, worse from change of weather. There are also tensive pains in the legs and arms, especially in the tendo Achillis, which feels as if shortened, drawn up.

Actæa may be used in cerebro-spinal meningitis, with great soreness and sensitiveness of the spine, the characteristic mental symptoms being present. It has been thought of in cases of old drunkards, or affections following a spree; in delirium tremens.

In this remedy as in many others useful in hysteria, there is profuse urination—great quantities of clear unrine passed—which seem to prostrate.

I append the following case:

September 29th, 1894, Mrs. George S——, age 43. Slight little woman, blue eyes, pale, bloodless, "feeling perfectly miserable."

Has had seven children in eight years, never can nurse them. Now seven and a half months pregnant. Generally of a cheerful disposition, but now depressed; seems under a constant gloom; constant fear of the pain of her confinement. Heartburn whenever pregnant. Starts in the stomach and rises to throat. It is worse before midnight and worse from drinking water.

Cramps in walls of abdomen as if knives were cutting her.

Worse after midnight. Better walking and rubbing. Feet fidgety.

Fœtal movements violent. Feels as if fœtus would push through abdominal walls. Fond of salt, aversion to pepper. Weak and worn out with child-bearing. Sensation as if tadpoles danced before eyes. Water disagrees; she never cares to drink it. She feels better in the open air. Wants but little cover at night at any time of the year. Suffered with chilblains before her marriage. Rapid pulse. Aversion to noise. Remedy, Actæa-racemosa, CM.

She told me later: "That medicine acted like a charm; I felt perfectly well until confined."

